# Hydrogen Sulfide Attenuates the Cognitive Dysfunction in Parkinson's Disease Rats via Promoting Hippocampal Microglia M2 Polarization by Enhancement of Hippocampal Warburg Effect

**DOI:** 10.1155/2022/2792348

**Published:** 2022-01-04

**Authors:** Qing Tian, Hui-Ling Tang, Yi-Yun Tang, Ping Zhang, Xuan Kang, Wei Zou, Xiao-Qing Tang

**Affiliations:** ^1^Hengyang Key Laboratory of Neurodegeneration and Cognitive Impairment, Institute of Neuroscience, Hengyang Medical School, University of South China, Hengyang, 421001 Hunan, China; ^2^The Affiliated Nanhua Hospital, Department of Neurology, Hengyang Medical School, University of South China, Hengyang, 421001 Hunan, China; ^3^The First Affiliated Hospital, Institute of Neurology, Hengyang Medical School, University of South China, Hengyang, 421001 Hunan, China

## Abstract

Identification of innovative therapeutic targets for the treatment of cognitive impairment in Parkinson's disease (PD) is urgently needed. Hydrogen sulfide (H_2_S) plays an important role in cognitive function. Therefore, this work is aimed at investigating whether H_2_S attenuates the cognitive impairment in PD and the underlying mechanisms. In the rotenone- (ROT-) established PD rat model, NaHS (a donor of H_2_S) attenuated the cognitive impairment and promoted microglia polarization from M1 towards M2 in the hippocampus of PD rats. NaHS also dramatically upregulated the Warburg effect in the hippocampus of PD rats. 2-Deoxyglucose (2-DG, an inhibitor of the Warburg effect) abolished NaHS-upregulated Warburg effect in the hippocampus of PD rats. Moreover, the inhibited hippocampal Warburg effect by 2-DG abrogated H_2_S-excited the enhancement of hippocampal microglia M2 polarization and the improvement of cognitive function in ROT-exposed rats. Our data demonstrated that H_2_S inhibits the cognitive dysfunction in PD via promoting microglia M2 polarization by enhancement of hippocampal Warburg effect.

## 1. Introduction

Parkinson's disease (PD), characterized by the loss of dopaminergic neurons, is a common neurodegenerative disease [1, 2]. PD is defined primarily as a movement disorder, with the typical dopamine-related motor symptoms being resting tremor, rigidity, bradykinesia, and postural instability. However, a wide variety of nonmotor symptoms are commonly observed in patients with PD [3]. The cognitive dysfunction is a frequent important nonmotor symptom encountered in PD patients [3]. It has been reported that the lesioned hippocampus, a temporal lobe structure, leads to memory dysfunction [4, 5]. Furthermore, aerobic exercise training is effective at reversing hippocampal volume loss, which is accompanied by improved memory function [6]. As reported that verbal memory deficient in recall and recognition is associated with atrophy of the hippocampus in newly diagnosed PD patients [7], recent advancement also observed hippocampal subfield atrophy of CA1 in participants who progressed to PD dementia [8]. All of these reflect an intimate relationship between hippocampal dysfunction and cognitive impairment in PD. However, there is no effective strategy to prevent the cognitive impairment in PD. Hydrogen sulfide (H_2_S), well known as a neuromodulator [9], alleviates cognitive deficits caused by multiple risk factors [10–12]. Our previous work demonstrated that the cognitive impairment in formaldehyde- or homocysteine-exposed rats is related to the disturbance of endogenous H_2_S [13, 14] and that supplementation of exogenous H_2_S prevents the cognitive impairment in homocysteine- [15, 16], chronic restrain stress- [17], and streptozotocin- [18] exposed rats. Collectively, the antagonistic action of H_2_S on cognitive dysfunction is widely recognized. Thus, it seems reasonable to speculate the therapeutic potential of H_2_S for the cognitive deficit in PD.

Microglia is the primary immune cells in the brain, acting as immune surveillance and host defense. Accumulating evidence has reported that microglia regulate neurodevelopment and synaptic plasticity [19, 20]. Like macrophages, microglia are designated classically activated (M1) and alternatively activated (M2) phenotypes [21]. M1 microglia release proinflammatory cytokines to destroy neurons, and contrarily, M2 microglia secrete neuroprotective mediators and trophic factors. It has been confirmed that promoting microglia M2 polarization enhances *α*-synuclein clearance in PD [22]. Furthermore, maintaining the balance of M1 and M2 is crucial to alleviate cognitive disorder in schizophrenia [23] and enhancing M2 polarization mitigates A*β*-caused cognitive dysfunction [24]. Additionally, H_2_S exerts a regulatory role in microglia polarization [25, 26]. Thus, to understand the mechanism underlying H_2_S-promoted cognitive function in PD, we investigated whether H_2_S skews M1 microglia polarization toward M2 activation.

The Warburg effect, also known as aerobic glycolysis, involves the metabolism of glucose to supply ATP and secrete lactate via glycolysis under aerobic conditions. Otto Warburg's work first reported that cancer cells have a propensity to metabolize glucose via glycolysis despite oxygen availability [27]. Interestingly, the Warburg effect also plays vital regulatory roles in the function of the brain, supporting the molecular demands of neuronal proliferation in embryonic development, promoting the maturational changes of neurons in postnatal development, and is responsible for changes at the synapse in adulthood [28, 29]. Moreover, lactate, a metabolite of the Warburg effect, exerts a regulatory role in synaptic plasticity by enhancing the expressions of Egr-1 and c-Fos. A recent study reported that lactate is required for long-term potentiation (LTP) formation [30], providing a new insight into the mechanism of cognition [31, 32]. These observations prompted us to raise the question of whether the Warburg effect mediates the antagonistic role of H_2_S in the cognitive deficit in PD.

In this study, we found that H_2_S alleviated the cognitive impairment, promoted hippocampal microglia M2 polarization, and enhanced hippocampal Warburg effect in the rotenone-induced PD rats. Furthermore, inhibition of the hippocampal Warburg effect abrogated H_2_S-exerted protection against the cognitive disorder and promotion of hippocampal microglia M2 polarization in PD rats. Our findings demonstrated that H_2_S enhances hippocampal Warburg effect, leading to promote microglia M2 polarization, which in turn prevents the cognitive disorder in PD.

## 2. Materials and Methods

### 2.1. Reagents

NaHS, rotenone (ROT), 2-deoxyglucose (2-DG), sunflower seed oil, and a lactate assay kit were purchased from Sigma (MO, USA). The primary antibodies against hexokinase II (HKII), pyruvate kinase M2 (PKM2), pyruvate dehydrogenase kinase 1 (PDK-1), pyruvate dehydrogenase (PDH), lactate dehydrogenase kinase A (LDHA), and arginase-1 (Arg-1) were obtained from Cell Signaling Technology (MA, USA). The primary antibodies against chitinase 3-like 3 (Ym-1) and inducible nitric oxide synthase (iNOS) and the Griess assay kit were obtained from Abcam (CA, USA). The Rat TGF-*β*1 ELISA kit was purchased from BioVision (CA, USA), and the Rat IL-4 ELISA Kit and Rat IL-1*β* ELISA Kit were purchased from ABclonal (Wuhan, China).

### 2.2. PD Animal Model and Animal Dosing

**Figure 1:**
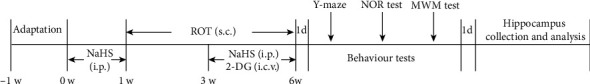
Schematic of experimental design. i.p.: intraperitoneal; s.c.: subcutaneous; i.c.v.: intracerebroventricular.

The adult male Sprague-Dawley (SD) rats weighing 260–280 g were obtained from SJA Lab. Each group contained 15 rats and each rat was housed individually with free access to food and water. The room humidity was controlled at 40%–46%, and the temperature was controlled within 22–26 °C. SD rats were exposed to ROT (2 mg/kg, s.c.) for 5 w to mimic the PD model [33]. After habituating to this new housing environment for 1 w, rats were administered NaHS (30 and 100 *μ*mol/kg, i.p.) for 1 w. Subsequently, rats were exposed to ROT via subcutaneous injection for 5 w and NaHS for 3 w simultaneously. In the 3^rd^ week of injecting ROT, rats were cotreated with 2-DG (3 *μ*g/w, i.c.v.) for 3 w. After performing behavioral tests, the brains of rats were collected under euthanasia (Figure 1). All experiments strictly complied with the *Guide for the Care and Use of Laboratory Animals* of the National Institutes of Health and were approved by the Animal Use and Protection Committee of the University of South China.

### 2.3. Intracerebroventricular Injection

Under deep anesthesia using pentobarbital sodium (45 mg/kg), animals were fixed on the stereotaxic apparatus for the following operation. The hair from the top of the skull was removed, fully exposing the bregma. The position of the right lateral ventricle was determined according to the following coordinates: AP, −1.0 mm; ML, −2.0 mm; DV, −4.0 mm. Then, the aseptic cannula was implanted into this location for drug administration. After surgery, penicillin was administered for 3 consecutive days to prevent infection. Rats were recovered for 1 w. 2-DG was administered into the lateral ventricle along the cannula using a microsyringe. The needle was removed slowly and kept halfway in for an extra 2 min to ensure complete delivery of the drug.

### 2.4. Y-Maze Test

The Y-maze test was used to evaluate the learning and memory of rats. The experimental apparatus consisted of three identical labeled arms (A, B, and C) at equal angles. At the beginning of the test, the rat was placed in the intersection of the three arms to explore freely for 5 min. The sequence and number of the rat entries into each arm were recorded. The rat entering different arms in turn during three consecutive chances was marked as a correct sequence (for example, ABC, BAC, or ACB, but not ABA and etc.). Once the rat completed the test, the three arms and the bottom of the apparatus were swept and wiped using 70% alcohol to eliminate the potential influence of odors and residues on experimental data. The alternation performance was calculated to evaluate the spatial differentiation memory of rats.

### 2.5. Novel Object Recognition (NOR) Test

The open-field box that was used in this experiment was 50 cm × 40 cm × 30 cm. To avoid being pushed or bitten by rats, the recognized objects were cylinders and tubes with a certain weight as well as hardness. The whole experiment was divided into three periods: the adaptation period, training period, and testing period. The adaptation period lasted for 2 d. All rats were introduced to the test box from the fixed position and were adapted to this new environment for 5 min without objects. During the training period, the rat entered the box with two identical objects from a fixed position, and the time that the rat spent exploring each of the two objects was recorded. After an hour-long retention interval, the rat was reintroduced to the test box, with one familiar object replaced by one novel object, and the time that the rat spent exploring each of the two objects over the course of 5 min was recorded. Touching, sniffing, and direct attention to an object rather than climbing or chewing objects were defined as exploratory behaviors. A distance between the nose and object within 2 cm was also regarded as exploratory behavior. The discrimination index was calculated to reflect the cognitive function of the rat, which was defined by the following equation: discrimination index = [(time spent on familiar object − time spent on novel object)/(time spent on familiar object + time spent on novel object)] × 100.

### 2.6. Morris Water Maze (MWM) Test

The MWM system mainly includes a circular pool with water and a hidden platform. The pool was divided into four quadrants, and the target quadrant was defined as the platform positioned in the center of this area. In the acquisition phase (days 1–5), rats were randomly introduced into the pool from different quadrants to search for the hidden platform in four trials per day with a 20-minute interval. The maximum swimming time for each rat was 120 s in each trial; otherwise, rats were guided to the hidden platform and allowed to stay there for 20 s. The swimming route and latency to the platform were tracked by the MT-200 Morris Image Motion System. In the probe phase (day 7), rats were reintroduced to the pool without a platform from the opposite position to the target quadrant. The time spent swimming in the target quadrant and the number of crossing the target platform of rats were recorded over the course of 120 s. Following the probe test, rats were subjected to the visible platform test to rule out possible deficits in vision and motor function, which may impact experimental data. The platform was replaced opposite to the target quadrant and was 2 cm higher above the water. Rats were reintroduced to the pool from the side opposite to the platform, and the escape latency and average speed were recorded.

### 2.7. Western Blot Analysis

After all behavioral tests were completed, rats were sacrificed, and hippocampal tissues of the brain were dissected. Then, hippocampal tissues were homogenized using lysis buffer, and supernatant was collected after centrifugation. The protein content was measured by a BCA protein assay kit. The prepared samples were added into sodium dodecyl sulfate-polyamide gel for electrophoresis and subsequently transferred to the polyvinylidene fluoride membrane. The membranes were blocked using 5% skim milk in Tris-buffered saline containing 0.1% Tween-20 (TBST). Two hours later, these were incubated with primary antibodies overnight at 4°C and subsequently washed 10 min in TBST followed by incubation with corresponding secondary antibodies for 2 h. Finally, membranes were visualized by an enhanced chemiluminescence system (BeyoECL Plus kit, Beyotime). AlphaImager 2200 software was used to calculate the signal of immunoblot.

### 2.8. Determination of Lactate Level in the Hippocampus

The level of lactate was analyzed by a lactate assay kit. In this assay, the lactate content was determined by a colorimetric product in an enzymatic assay. Briefly, hippocampal tissues were homogenized in 4 volumes of the Lactate Assay Buffer and centrifuged at 13,000 × *g* for 10 min. Supernatants were collected and deproteinized to remove lactate dehydrogenase. Subsequently, 3 *μ*l of each sample was added into a 96-well plate, and Lactate Assay Buffer was supplemented to a final volume of 50 *μ*l per well. After that, 50 *μ*l of Master Reaction Mix (46 *μ*l Lactate Assay Buffer, 2 *μ*l Lactate Enzyme Mix, and 2 *μ*l Lactate Probe) was added to each well and incubated at room temperature without light. Thirty minutes later, the absorbance at 450 nm was measured under a microplate reader. The content of lactate in samples was determined by a formula and a standard curve referencing the manufacturer's instructions.

### 2.9. Immunofluorescence Staining

After fixing in 4% paraformaldehyde, brain samples were dehydrated and subsequently embedded into paraffin. The paraffin-embedded sections were treated with citric acid-containing buffer and coated in 3% H_2_O_2_ for 10 min at 37°C. Following blockages to eliminate nonspecific staining, sections were incubated with the primary antibodies at 4°C overnight. After washing in PBS three times for 5 min, sections were incubated with corresponding secondary antibodies in the dark at 37°C to visualize the fluorescent signal. The nuclei were visualized using DAPI staining solution for 1 h without light. While coverslips were mounted, stained sections were visualized and photographed under a microscope.

### 2.10. Enzyme-Linked Immunosorbent Assay (ELISA)

The contents of TGF-*β*1, IL-1*β*, and IL-4 were detected by the corresponding ELISA kits. According to the manufacturer's instructions, supernatants of tissues were collected via lysis and centrifugation. Samples were added into the microplate stripe wells and then mixed with HRP-labeled antibody. Microplate was coated with microplate sealers and incubated at 37°C. After washing, substrate A and substrate B were added separately to each well to incubate in the dark and then supplemented with stop solution. The optical density at 450 nm was measured within 15 min using a microplate reader.

### 2.11. Griess Assay

The level of NO was detected using a Griess assay kit. Supernatant was collected and added into a 96-well plate. Solution I and solution II were added, mixed, and then incubated for 20 min without light. The optical density at 540 nm was measured using a microplate reader.

### 2.12. Statistical Analysis

All data in this paper were obtained using SPSS 20 software and described as the mean ± S.E.M. The significance of intergroup differences was determined by one-way ANOVA. The variance and multiple comparisons were evaluated by LSD-*t*. *P* < 0.05 was set as the standard for a significant difference.

## 3. Results

### 3.1. H_2_S Attenuates the Cognitive Impairment in ROT-Induced PD Rats

We explored the effect of H_2_S on the cognitive disorder in ROT-induced PD rats using the Y-maze, NOR, and MWM tests.

**Figure 2:**
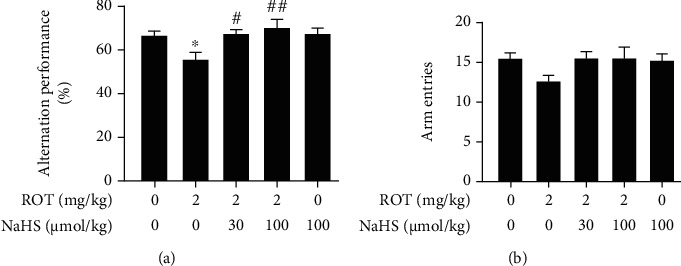
Effect of H_2_S on the cognitive impairment of ROT-induced PD rats in the Y-maze test. After pretreatment with NaHS (30 and 100 *μ*mol/kg, i.p.) for 1 w, SD rats were injected with ROT (2 mg/kg, s.c.) for 5 w and NaHS for 3 w simultaneously. All rats were subjected to the Y-maze test. The alternation performance (a) and the arm entries (b) over the course of 5 min were analyzed. Values are presented as the mean ± S.E.M. (*n* = 9–15). ^∗^*P* < 0.05 vs. control group; ^#^*P* < 0.05 and ^##^*P* < 0.01 vs. ROT treatment alone group.

In the Y-maze test, NaHS (a donor of H_2_S) markedly increased the alternation performance in the ROT-exposed rats (Figure 2(a)). In addition, there was no significant difference in the number of total entries among experimental groups (Figure 2(b)).

**Figure 3:**
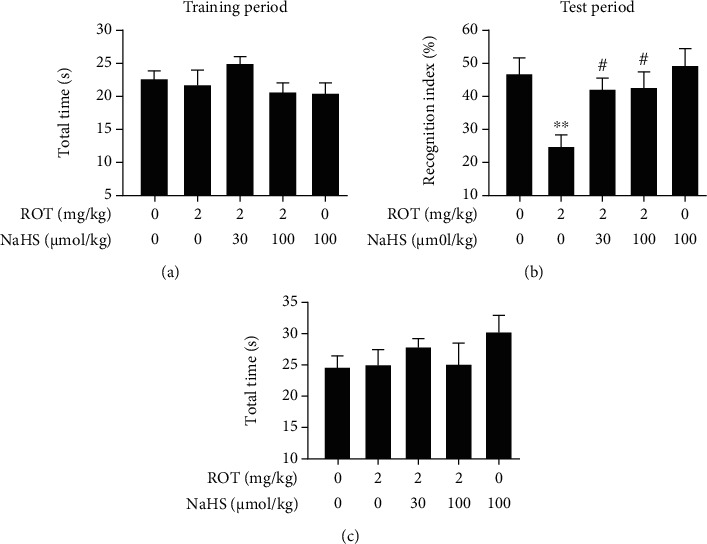
Effect of H_2_S on the cognitive impairment of ROT-induced PD rats in the NOR test. After pretreatment with NaHS (30 and 100 *μ*mol/kg, i.p.) for 1 w, SD rats were injected with ROT (2 mg/kg, s.c.) for 5 w and NaHS for 3 w simultaneously. Rats were subjected to the NOR test. The total time in the training period (a) and the recognition index (b) as well as total time (c) in the test period over the course of 5 min were assessed. Values are presented as the mean ± S.E.M. (*n* = 9–15). ^∗∗^*P* < 0.01 vs. control group; ^#^*P* < 0.05 vs. ROT exposure alone group.

In the NOR test, there was no significant difference in the total time spent exploring the two objects among the five groups during the training phase (Figure 3(a)) as well as testing phase (Figure 3(c)). However, the discrimination index in the testing phase was decreased in the ROT-exposed rats and dramatically elevated by treatment with NaHS (Figure 3(b)).

**Figure 4:**
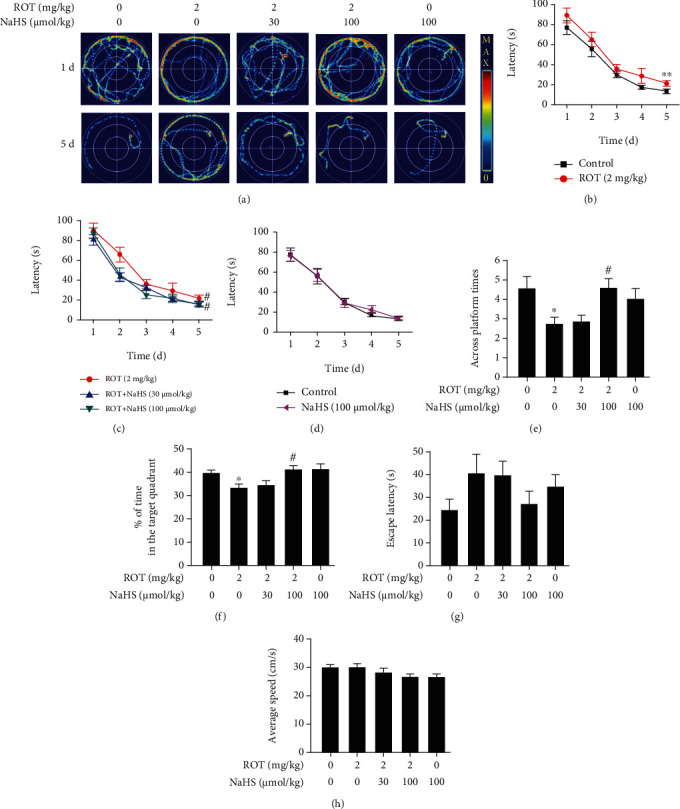
Effect of H_2_S on the cognitive impairment of ROT-induced PD rats in the MWM test. After pretreated with NaHS (30 and 100 *μ*mol/kg, i.p.) for 1 w, SD rats were injected with ROT (2 mg/kg, s.c.) for 5 w and NaHS for 3 w simultaneously. Rats were then subjected to the MWM test. The representative swimming routes of one rat for each group in searching for the platform were recorded on the 1^st^ and 5^th^ training days (a). The latency to find the platform in the acquisition phase was recorded (b–d). The number of crossing the platform (e) and the percentage of time spent in the target quadrant (f) in the probe trial phase were assessed. The escape latency (g) and average speed (h) of rats in the visible platform phase were recorded. Values are presented as the mean ± S.E.M. (*n* = 9 − 15). ^∗^*P* < 0.05 and ^∗∗^*P* < 0.01 vs. control group; ^#^*P* < 0.05 vs. ROT exposure alone group.

The MWM test was also used to explore the effect of H_2_S on the declined learning and memory in the ROT-induced PD rats. In the acquisition phase of the MWM test, the swimming routes for searching for the platform were tortuous and complicated for all groups on the first training day, while NaHS significantly simplified the search routes of ROT-exposed rats on the fifth day (Figure 4(a)). Furthermore, ROT treatment lengthened the latency to find the platform compared to the control group (Figure 4(b)), which was significantly reduced by NaHS supplementation (Figure 4(c)). NaHS treatment alone showed no impact on the normal rats to find the platform (Figure 4(d)). In the probe trial phase of the MWM test, NaHS significantly increased the number across the target platform (Figure 4(e)) and the time spent in the target quadrant (Figure 4(f)) among the ROT-exposed rats. In the visible platform phase of the MWM test, no significant differences in the escape latency (Figure 4(g)) and the average speed (Figure 4(h)) were observed among groups.

Together, these findings indicated that H_2_S attenuates the cognitive impairment in the ROT-induced PD rats.

### 3.2. H_2_S Promotes Microglia Polarization from M1 to M2 in the Hippocampus of ROT-Exposed Rats

**Figure 5:**
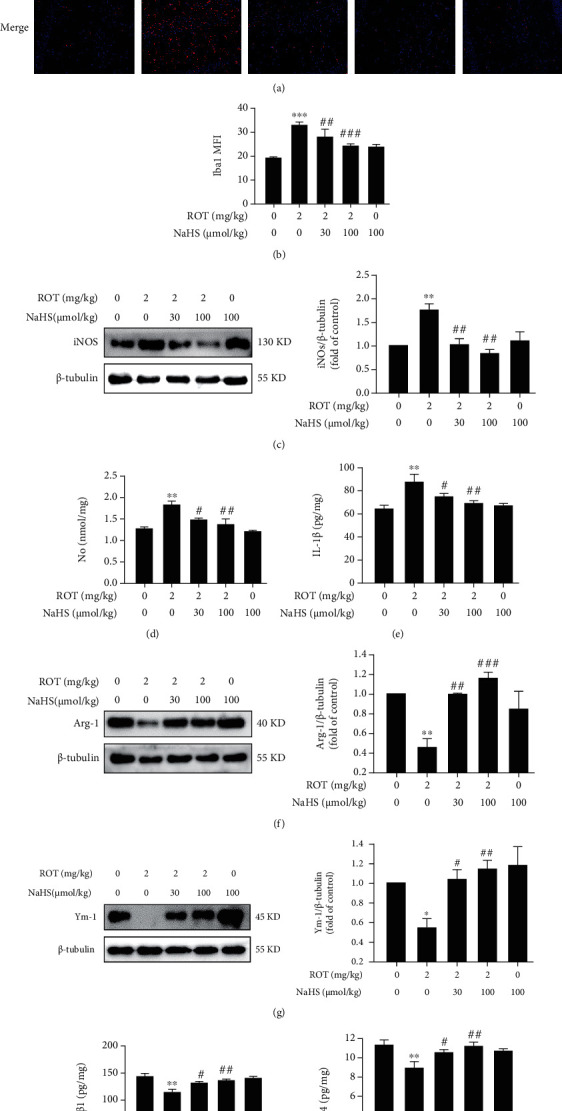
Effect of H_2_S on microglia polarization in the hippocampus of ROT-induced PD rats. After pretreatment with NaHS (30 and 100 *μ*mol/kg, i.p.) for 1 w, SD rats were injected with ROT (2 mg/kg, s.c.) for 5 w and NaHS for 3 w simultaneously. Representative images of activated microglia (a). Quantification of mean fluorescence intensity (MFI) of Iba1 (b). The nuclei were stained by DAPI (blue), and the activated microglia was stained by Iba1 (red). Magnification, 20x. The expressions of iNOS (c), Arg-1 (f), and Ym-1 (g) were measured by western blot. The level of NO (d) was measured by the Griess assay kit. The contents of IL-1*β* (e), TGF-*β*1 (h), and IL-4 (i) were detected by ELISA. Values are presented as the mean ± S.E.M. (*n* = 3). ^∗^*P* < 0.05, ^∗∗^*P* < 0.01, and ^∗∗∗^*P* < 0.001 vs. control group; ^#^*P* < 0.05, ^##^*P* < 0.01, and ^###^*P* < 0.001 vs. ROT treatment alone group.

Next, we assessed the effect of H_2_S on microglia polarization in the hippocampus of ROT-exposed rats. The bright fluorescence of Iba1 in ROT-exposed rats exhibited a reduced state after NaHS treatment (Figures 5(a) and 5(b)), indicating the inhibitory role of H_2_S in microglia activation. The most important characteristic of microglia polarization is the phenotype alteration. We found that H_2_S inhibited M1 polarization of microglia, which was characterized by the decreased expression of iNOS (Figure 5(c)) and the secretions of NO (Figure 5(d)) and IL-1*β* (Figure 5(e)) in the hippocampus of rats cotreatment with ROT and NaHS. More strikingly, H_2_S accelerated microglia polarization to M2 phenotype, featured with the upregulated protein expressions of Arg-1 (Figure 5(f)) as well as Ym-1 (Figure 5(g)) and the increased secretions of TGF-*β*1 (Figure 5(h)) as well as IL-4 (Figure 5(i)) in the hippocampus of rats cotreatment with ROT and NaHS. These data indicated that H_2_S promotes microglia M2 polarization in the hippocampus of ROT-induced PD rats.

### 3.3. H_2_S Upregulates the Hippocampal Warburg Effect in the ROT-Exposed Rats

**Figure 6:**
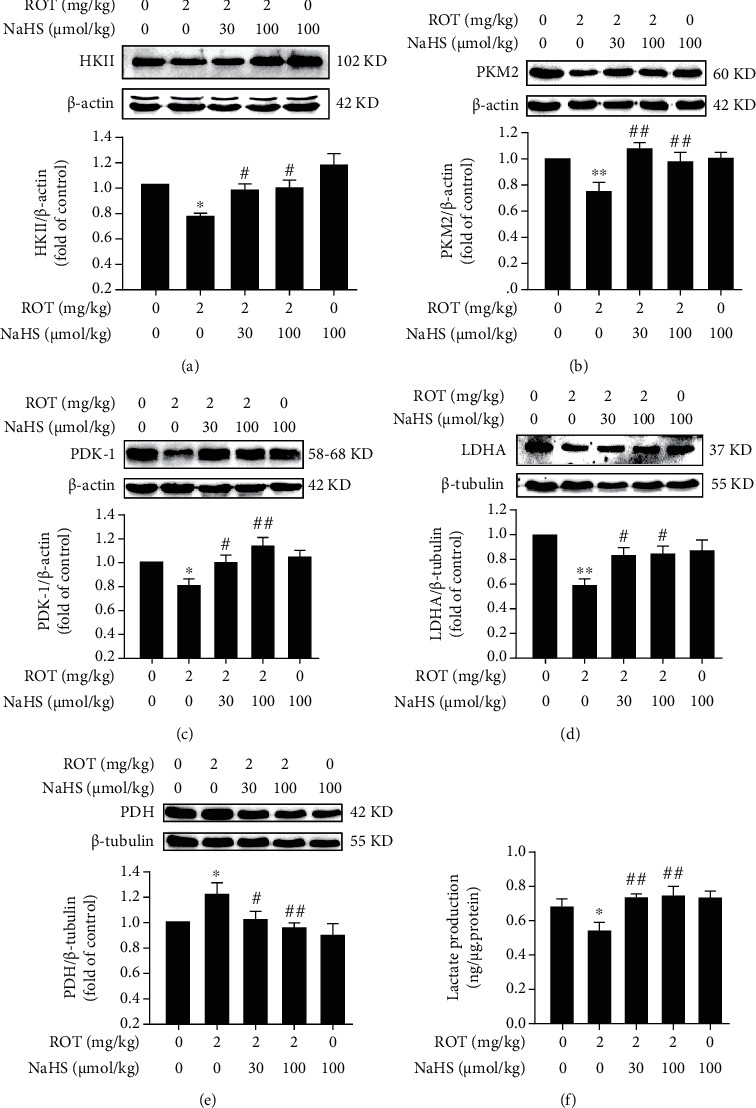
Effect of H_2_S on the Warburg effect in the hippocampus of ROT-induced PD rats. After pretreatment with NaHS (30 and 100 *μ*mol/kg, i.p.) for 1 w, SD rats were injected with ROT (2 mg/kg, s.c.) for 5 w and NaHS for 3 w simultaneously. The expressions of HKII (a), PKM2 (b), PDK-1 (c), LDHA (d), and PDH (e) in the hippocampus were detected by western blot. The content of lactate (f) in the hippocampus was tested by a lactate assay kit. Values are presented as the mean ± S.E.M. (*n* = 3). ^∗^*P* < 0.05 and ^∗∗^*P* < 0.01 vs. control group; ^#^*P* < 0.05 and ^##^*P* < 0.01 vs. ROT treatment alone group.

To investigate the role of H_2_S in the hippocampal Warburg effect of rats, we measured the hallmarks related to the Warburg effect in the hippocampus. NaHS markedly upregulated the expressions of HKII (Figure 6(a)), PKM2 (Figure 6(b)), PDK-1 (Figure 6(c)), and LDHA (Figure 6(d)), as well as downregulated the expression of PDH (Figure 6(e)) in the hippocampus of ROT-exposed rats. Moreover, the content of lactate decreased in the hippocampus of ROT-treated rats, which was increased by treatment with NaHS (Figure 6(f)). Taken together, these findings indicated that H_2_S enhances the Warburg effect in the hippocampus of ROT-treated rats.

### 3.4. 2-DG Prevents the Upregulatory Role of H_2_S in the Hippocampal Warburg Effect of ROT-Exposed Rats

**Figure 7:**
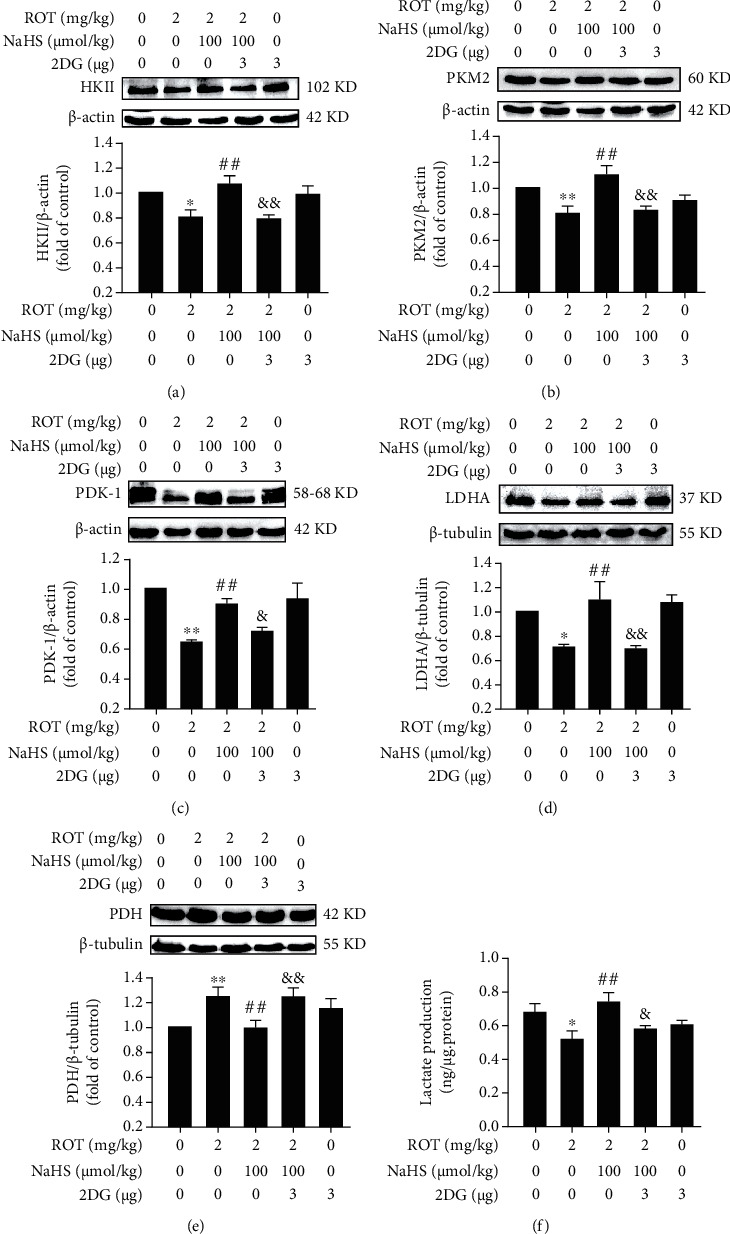
Effect of 2-DG on H_2_S-enhanced hippocampal Warburg effect in ROT-treated rats. After pretreatment with NaHS (100 *μ*mol/kg, i.p.) for 1 w, SD rats were injected with ROT (2 mg/kg, s.c.) for 5 w. In the 3^rd^ week of injecting ROT, rats were cotreated with NaHS (100 *μ*mol/kg, i.p.) and 2-DG (3 *μ*g/w, i.c.v.) for 3 w simultaneously. The expressions of HKII (a), PKM2 (b), PDK-1 (c), LDHA (d), and PDH (e) in the hippocampus were detected by western blot. The content of lactate (f) in the hippocampus was tested by a lactate assay kit. Values are presented as the mean ± S.E.M. (*n* = 3). ^∗^*P* < 0.05 and ^∗∗^*P* < 0.01 vs. control group; ^##^*P* < 0.01 vs. ROT treatment alone group; ^&^*P* < 0.05 and ^&&^*P* < 0.01 vs. ROT and NaHS cotreatment groups.

To investigate whether inhibited the hippocampal Warburg effect reverses the protection of H_2_S against ROT-induced decline in cognitive function, we first investigated whether 2-DG (an inhibitor of the Warburg effect) [34, 35] abolished H_2_S-enhanced Warburg effect in the hippocampus of ROT-exposed rats. 2-DG dramatically ameliorated NaHS-elicited upregulation in the expressions of HKII (Figure 7(a)), PKM2 (Figure 7(b)), PDK-1 (Figure 7(c)), and LDHA (Figure 7(d)) as well as downregulation in the expression of PDH (Figure 7(e)) in the hippocampus of ROT-exposed rats. Furthermore, 2-DG significantly reversed the role of NaHS in enhancing the hippocampal lactate level of ROT-exposed rats (Figure 7(f)). These data indicated that 2-DG prevents H_2_S-enhanced Warburg effect in the hippocampus of ROT-induced PD rats.

### 3.5. Inhibited Hippocampal Warburg Effect Reverses H_2_S-Attenuated Cognitive Dysfunction of PD Rats

To confirm the mediatory role of hippocampal Warburg effect in H_2_S-promoted cognitive function of PD rats, we further explored whether inhibited hippocampal Warburg effect by 2-DG abolishes H_2_S-promoted cognitive function in the ROT-treated rats.

**Figure 8:**
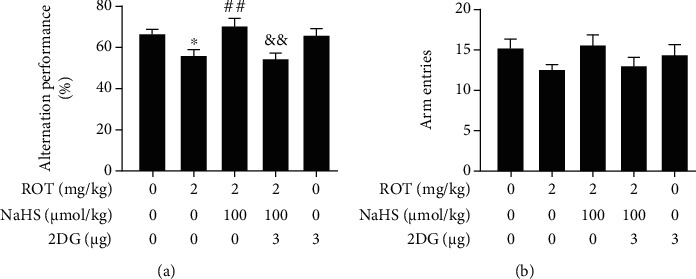
Effect of 2-DG on H_2_S-ameliorated cognitive impairment of ROT-exposed rats in the Y-maze test. After pretreatment with NaHS (100 *μ*mol/kg, i.p.) for 1 w, SD rats were injected with ROT (2 mg/kg, s.c.) for 5 w. In the 3^rd^ week of ROT injection, rats were cotreated with NaHS (100 *μ*mol/kg, i.p.) and 2-DG (3 *μ*g/w, i.c.v.) for 3 w simultaneously. All rats were subjected to the Y-maze test. The alternation performance (a) and arm entries (b) over the course of 5 min were analyzed. Values are presented as the mean ± S.E.M. (*n* = 9–15). ^∗^*P* < 0.05 vs. control group; ^##^*P* < 0.01 vs. ROT treatment alone group; ^&&^*P* < 0.01 vs. ROT and NaHS cotreatment groups.

In the Y-maze test, 2-DG dramatically reversed the upregulatory effect of NaHS on the alternative performance of ROT-treated rats (Figure 8(a)). The total entries showed no significant difference among the groups (Figure 8(b)).

**Figure 9:**
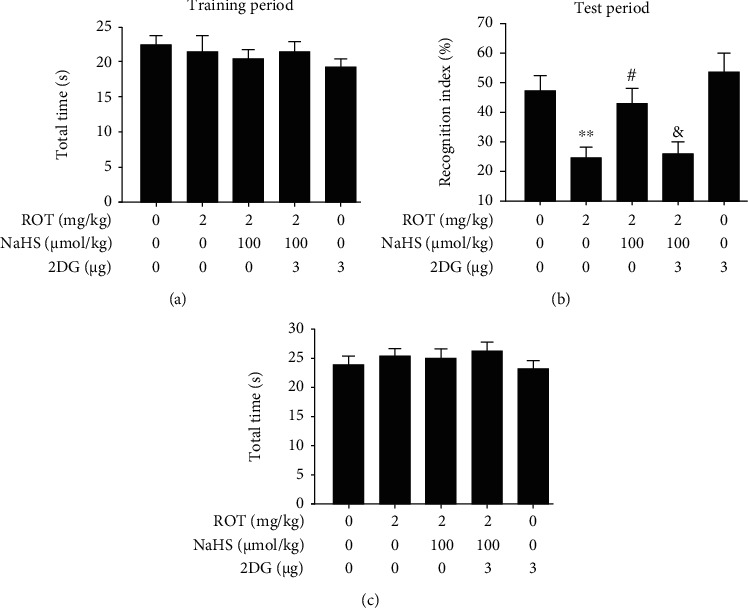
Effect of 2-DG on H_2_S-ameliorated cognitive impairment of ROT-induced PD rats in the NOR test. After pretreatment with NaHS (100 *μ*mol/kg, i.p.) for 1 w, SD rats were injected with ROT (2 mg/kg, s.c.) for 5 w. In the 3^rd^ week of ROT injection, rats were cotreated with NaHS (100 *μ*mol/kg, i.p.) and 2-DG (3 *μ*g/w, i.c.v.) for 3 w simultaneously. All rats were subjected to the NOR test. The total time in the training period (a) and recognition index (b) as well as the total time (c) in the test period over the course of 5 min were recorded. Values are presented as the mean ± S.E.M. (*n* = 9–15). ^∗∗^*P* < 0.01 vs. control group; ^#^*P* < 0.05 vs. ROT treatment alone group; ^&^*P* < 0.05 vs. ROT and NaHS cotreatment groups.

In the NOR test, 2-DG greatly decreased the recognition index of rats cotreated with ROT and NaHS (Figure 9(b)). There was no significant difference in the total time spent in exploring objects (Figures 9(a) and 9(c)).

**Figure 10:**
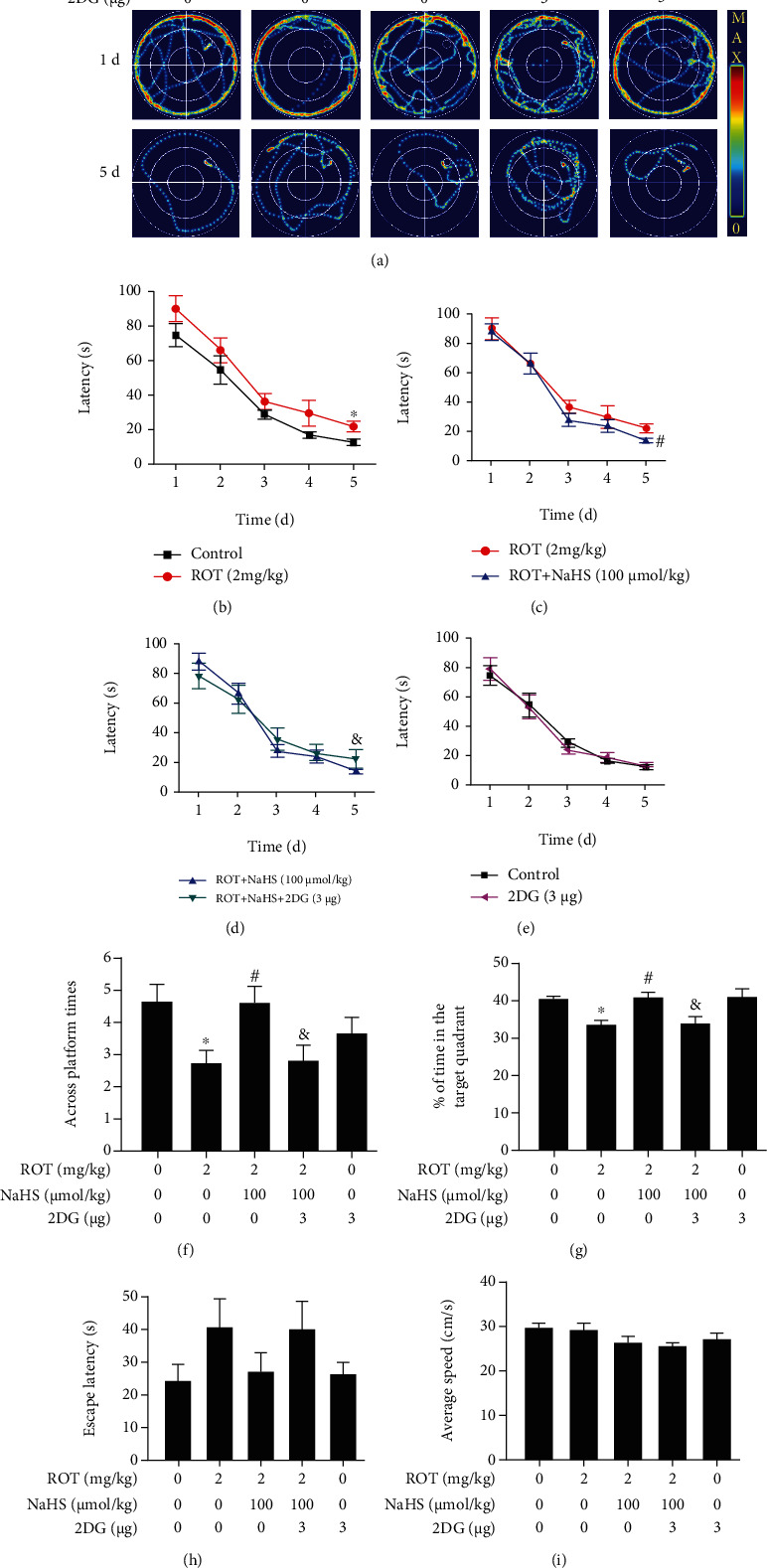
Effect of 2-DG on H_2_S-ameliorated cognitive dysfunction of ROT-induced PD rats in the MWM test. After pretreatment with NaHS (100 *μ*mol/kg, i.p.) for 1 w, SD rats were injected with ROT (2 mg/kg, s.c.) for 5 w. In the 3^rd^ week of ROT injection, rats were cotreated with NaHS (100 *μ*mol/kg, i.p.) and 2-DG (3 *μ*g/w, i.c.v.) for 3 w simultaneously. The representative swimming routes of one rat for each group in searching for the platform were recorded on the 1^st^ and 5^th^ training days (a). The latency to find the platform in the acquisition phase was recorded (b–e). The number across platform (f) and the percentage of time spent in the target quadrant (g) in the probe trial phase were recorded. The escape latency (h) and average speed (i) of rats in the visible platform phase were recorded. Values are presented as the mean ± S.E.M. (*n* = 9 − 15). ^∗^*P* < 0.05 vs. control group; ^#^*P* < 0.05 vs. ROT treatment alone group; ^&^*P* < 0.05 vs. ROT and NaHS cotreatment groups.

In the acquisition phase of the MWM test, 2-DG significantly complicated the swimming routes (Figure 10(a)) and extended the latency to find the platform in the rats cotreated with ROT and NaHS (Figures 10(b)–10(e)). In the probe trial of the MWM test, 2-DG decreased the number across the target platform (Figure 10(f)) and the percentage of time spent in the target quadrant (Figure 10(g)) of the rats cotreated with ROT and NaHS. The visible platform phase in the MWM test showed no significance in the escape latency (Figure 10(h)) or the average speed (Figure 10(i)) among all groups.

Taken together, suppression in the hippocampal Warburg effect abrogated the protection of H_2_S against the cognitive impairment in ROT-exerted PD rats.

### 3.6. Inhibited Hippocampal Warburg Effect Reverses H_2_S-Promoted Microglia M2 Polarization in the Hippocampus of ROT-Induced PD Rats

**Figure 11:**
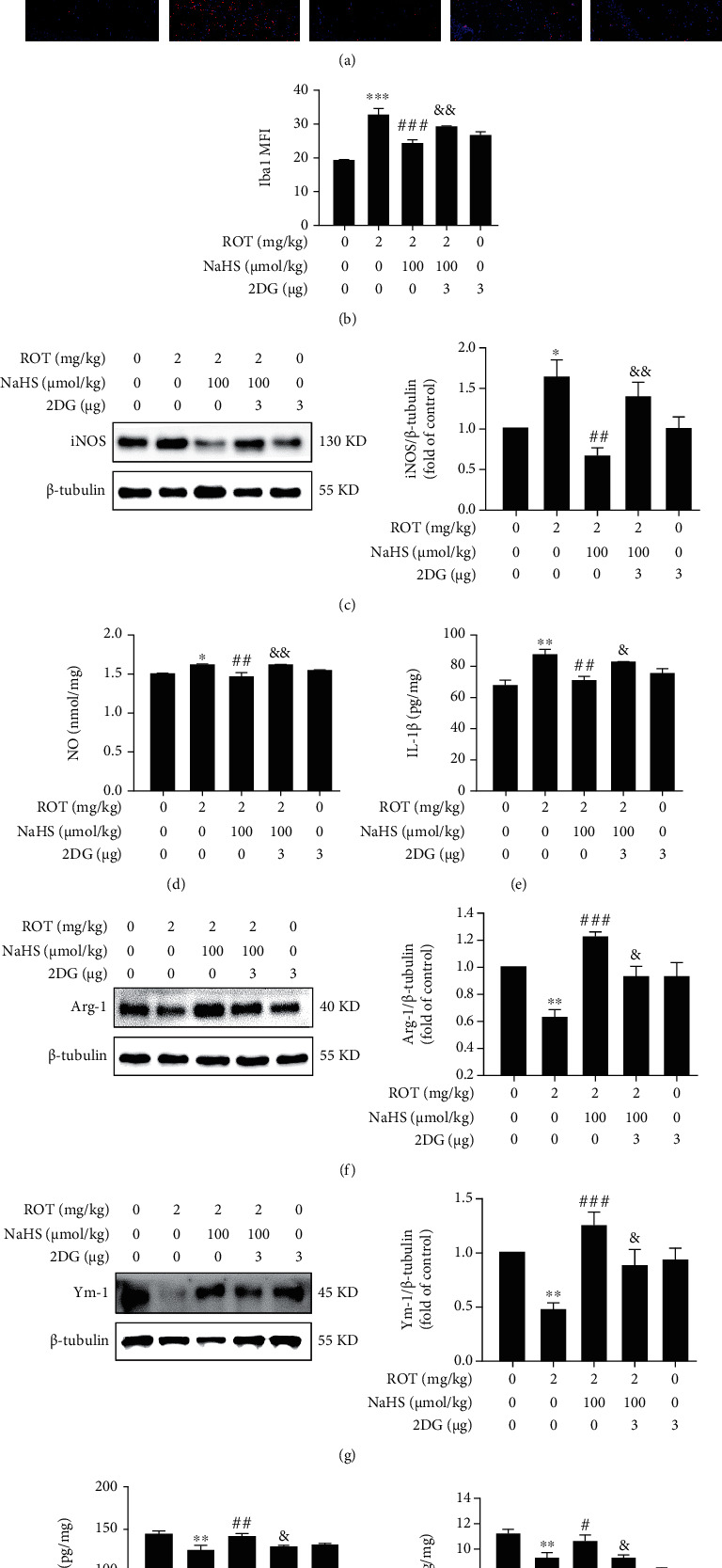
Effect of 2-DG on H_2_S-promoted microglia M2 polarization in ROT-exerted PD rats. After pretreatment with NaHS (100 *μ*mol/kg, i.p.) for 1 w, SD rats were injected with ROT (2 mg/kg, s.c.) for 5 w. On the 3^rd^ w of injecting ROT, rats were cotreated with NaHS (100 *μ*mol/kg, i.p.) and 2-DG (3 *μ*g/w, i.c.v.) for 3 w simultaneously. Representative images of activated microglia (a). Quantitation of mean fluorescence intensity (MFI) of Iba1 (b). The nuclei were stained by DAPI (blue), and the activated microglia was stained by Iba1 (red). Magnification, 20x. The expressions of iNOS (c), Arg-1 (f), and Ym-1 (g) were measured by western blot. The level of NO (d) was measured by the Griess assay kit. The contents of IL-1*β* (e), TGF-*β*1 (h), and IL-4 (i) were detected by ELISA. Values are presented as the mean ± S.E.M. (*n* = 3). ^∗^*P* < 0.05, ^∗∗^*P* < 0.01, and ^∗∗∗^*P* < 0.001 vs. control group; ^#^*P* < 0.05, ^##^*P* < 0.01, and ^###^*P* < 0.001 vs. ROT treatment alone group; ^&^*P* < 0.05 and ^&&^*P* < 0.01 vs. the ROT and NaHS cotreatment groups.

Next, we explored whether inhibited hippocampal Warburg effect by 2-DG reverses H_2_S-promoted microglia M2 polarization in the hippocampus of ROT-induced PD rats. Our data showed that 2-DG increased the fluorescence intensity of Iba-1 (Figures 11(a) and 11(b)) in the rats cotreated with ROT and NaHS, indicating that inhibited hippocampal Warburg effect by 2-DG restored the microglia activation. Additionally, 2-DG supplementation led to increases in the expression of iNOS (Figure 11(c)) as well as the contents of NO (Figure 11(d)) and IL-1*β* (Figure 11(e)) in the rats cotreated with ROT and NaHS, indicating that inhibited hippocampal Warburg effect by 2-DG enhanced M1 polarization. Conversely, 2-DG reduced the expressions of Arg-1(Figure 11(f)) and Ym-1 (Figure 11(g)) as well as the contents of TGF-*β*1 (Figure 11(h)) and IL-4 (Figure 11(i)) in the rats cotreated with ROT and NaHS, indicating inhibited hippocampal Warburg effect by 2-DG reduced M2 polarization. These results demonstrated that inhibited hippocampal Warburg effect abolishes H_2_S-accelerated microglia M2 polarization in the hippocampus of ROT-induced PD rats.

## 4. Discussion

How to effectively ameliorate cognitive impairment is a huge problem that needs to be solved to improve the life quality of PD patients. In this study, we demonstrated that H_2_S dramatically enhanced the learning and memory as well as the hippocampal microglia M2 polarization in the ROT-induced PD rats. H_2_S also promoted the hippocampal Warburg effect in the ROT-induced PD rats. Moreover, inhibition of the Warburg effect in the hippocampus significantly reversed H_2_S-promoted cognitive function and hippocampal microglia M2 polarization in the ROT-induced PD rats. Our findings identify H_2_S as a potential innovative inhibitor of cognitive deficit in PD and suggest that this effect is achieved via promoting microglia M2 polarization by upregulation of Warburg effect in the hippocampus.

PD is one of the most familiar neurodegenerative diseases in the clinic, featuring motor and nonmotor disorders [36, 37]. Dementia, a nonmotor symptom, develops in the early stage of PD. Increasing studies using animal models have verified that H_2_S recovers the number of dopaminergic neurons to normal and mitigates motor disorders [38, 39], identifying H_2_S as a new hope for the treatment of PD [40]. As a novel gaseous signaling molecule, H_2_S has the capacity to attenuate deficits in learning and memory induced by risk factors [16, 17, 41]. Accordingly, our study is aimed at investigating whether H_2_S protects against the cognitive impairment in ROT-induced PD rats. In the Y-maze test, we found that NaHS supplementation markedly increased alternation performance in ROT-exposed rats, suggesting a benefit of H_2_S to the cognitive function of ROT-exposed rats. In the NOR test, the declined recognition index in ROT-treated rats was reversed by the administration of NaHS, which indicated that H_2_S ameliorates deficits in learning and memory in ROT-treated rats. In the MWM test, NaHS greatly shortened the escape latency in the acquisition phase and increased the number of crossing platform as well as the time spent in the target quadrant in the probe trial of ROT-exposed rats, which further suggested that the impairment in spatial learning and memory in ROT-treated rats is reversed by H_2_S. Collectively, our data demonstrate that H_2_S prevents cognitive dysfunction in ROT-induced PD rats. It has demonstrated that H_2_S reduces neurodegeneration in MPP^+^-, ROT-, and 6-hydroxydopamine (6-OHDA)-caused PD models [42–44]. Thus, we believe that H_2_S holds promise for providing therapeutic benefits in the cognitive impairment of PD.

Microglia is the first line of defense of the central nervous system in the resting condition; microglia not only provide surveillance but also respond to danger signals [45]. Activated microglia exhibits two different statuses, M1 and M2. A previous study revealed microglia activation in the hippocampus of PD patients [46]. Furthermore, microglia play a crucial role in neuronal plasticity and neurogenesis [19, 47]. The hippocampus, one of limbic system structures, is well known in learning and memory. It also has been reported that hippocampus lesion contributes to memory dysfunction [4, 5], and reversing hippocampal volume loss improves memory function [6]. Cognitive impairment and hippocampal atrophy are hallmark features in PD patients, and hippocampal atrophy is reported to predict PD dementia [48, 49]. Microglia polarization is known to influence cognitive function and has therefore become a main player in neurodegenerative diseases leading to dementia. It has been reported that young offspring rats with cognitive impairment after maternal sleep deprivation exposure exhibit increase in microglia activation and M1 phenotype of microglia as well as a decrease in M2 phenotype of microglia [50]. Suppressing microglia M1 polarization in the hippocampus is an important mechanism in protecting against chronic unpredictable mild stress-induced cognitive disturbance [51]. Similarly, promoting microglia M2 polarization is able to ameliorate cognitive deficits in AD mice [52]. Inspiringly, inhibition of hippocampal microglial activation alleviates cognitive impairment in a PD model [53]. Therefore, the microglia polarization in the hippocampus was focused in the cognitive decline of PD. There are increased activation and M1 phenotype of microglia and decreased M2 phenotype of microglia in the hippocampus of ROT-treated rats. Therefore, we suggested that the cognitive deficit in PD model is also closely related to increased M1 polarization and reduced M2 polarization of microglia in the hippocampus. Notably, supplementation of NaHS significantly promoted hippocampal microglia M2 polarization in ROT-induced PD rats. Moreover, enhancing endogenous H_2_S via overexpression of cystathionine *β*-synthase promotes M2 polarization in lipopolysaccharide- [54] or ROT- [26] stimulated microglia. Similarly, exogenous H_2_S application with NaHS evokes the M2 polarization of ROT-exposed primary microglia [26]. Collectively, our data suggest the critical role of promoting hippocampal microglia M2 polarization in H_2_S-exerted protection against the cognitive dysfunction in PD. Microglia polarization occupies an important role in cognitive function and it is worth exploring deeply. Our future studies in this exciting and growing field will continue to reveal the effects of microglia in other brain regions on the cognitive impairment in PD.

The Warburg effect, or aerobic glycolysis, is an energy metabolism shift to glycolysis under aerobic conditions. This phenomenon was first discovered in cancer by Otto Warburg [55]. Besides supplying energy quickly, the Warburg effect serves as a vital characteristic of nontumor disease, especially in neurodegenerative disease [56, 57]. Increasing the expression of HKII attenuates ROT-induced cell death [58]. Corona et al. confirmed that an elevated Warburg effect in the brain reduces the amount of dopaminergic neuron death and improves PD symptoms [59]. More importantly, the Warburg effect maintains LTP and regulates synaptic reconstruction, resulting in an enhancement in learning and memory [30, 32]. Thus, there is a scientific research value to elucidate the mechanism underlying H_2_S-attenuated cognitive impairment in the ROT-exposed rats from the new perspective of the hippocampal Warburg effect. We found that the expressions of HKII, PKM2, PDK-1, and LDHA and the content of lactate in the hippocampus of ROT-treated rats were decreased, while the expression of PDH was increased. All these data indicated the downregulation of the hippocampal Warburg effect in ROT-exposed rats. Recent evidence confirmed that downregulation of Warburg effect via dichloroacetate impairs the learning and memory in mice [60], suggesting a requirement of Warburg effect for spatial memory acquisition. Furthermore, Shannon et al. also support the contribution of brain Warburg effect to learning [61]. In the present work, we demonstrated that H_2_S increased the hippocampal Warburg effect in ROT-exposed rats and that inhibited Warburg effect reversed the protection of H_2_S against cognitive dysfunction in ROT-exposed rats. Collectively, enhancing hippocampal Warburg effect plays a crucial role in H_2_S-provided therapeutic benefits in the cognitive impairment of PD.

Immune cells need different amounts of energy to exert various functions, and the metabolic pathways responsible for energy generation are determined by cellular phenotypes [62]. Under the classical inflammatory activation process (M1), microglia often undergo metabolic reprogramming toward aerobic glycolysis. In comparison, microglia with M2 status induced by IL-4 stimulation exhibit decreased lactate release and glucose consumption, suggesting reduced aerobic glycolysis [63, 64]. 2-DG, an inhibitor of HKII, is widely used to downregulate the Warburg effect [65], which was also confirmed in our study. More interestingly, 2-DG blunts the release of M1 polarization-associated markers TNF-*α* and IL-6 in primary microglia [66]. Given the link between microglia polarization and energy metabolism, whether the Warburg effect-mediated M2 microglia polarization contributes to H_2_S-produced neuroprotection in cognitive disorder is worth to research. From our data, 2-DG reversed the reduced M1 markers and the increased M2 markers in the hippocampal microglia by NaHS in ROT-induced PD rats, representing a microglia transition from M2 to M1. Additionally, 2-DG dramatically reversed H_2_S the attenuated cognitive deficits in the ROT-induced PD rats. In summary, our findings verified that H_2_S enhances the hippocampal Warburg effect to accelerate microglia polarization toward M2, which consequently ameliorates cognitive impairment.

Taken together, our findings demonstrated that H_2_S produces a protective response to the cognitive dysfunction of PD as a result of elevated microglia M2 polarization via enhancing the hippocampal Warburg effect. This study elucidates a previously unrecognized mechanism in the antagonistic effect of H_2_S on cognitive deficits and thereby provides a strong basis for the development of therapeutics targeting H_2_S bioavailability or the Warburg effect in the treatment of comorbid cognitive disorder and PD that is refractory to current drugs. Additionally, it is necessary to make effort to clarity the pathologic alteration in Parkinson's cognitive dysfunction. We will pay more attention to the relationship between pathologic alteration and microglia activation in our next step.

## Data Availability

The datasets analyzed during the current study are available from the corresponding authors upon reasonable request.
